# Fasting induces a biphasic adaptive metabolic response in murine small intestine

**DOI:** 10.1186/1471-2164-8-361

**Published:** 2007-10-09

**Authors:** Milka Sokolović, Diederik Wehkamp, Aleksandar Sokolović, Jacqueline Vermeulen, Lisa A Gilhuijs-Pederson, Rachel IM van Haaften, Yuri Nikolsky, Chris TA Evelo, Antoine HC van Kampen, Theodorus BM Hakvoort, Wouter H Lamers

**Affiliations:** 1 AMC Liver Centre, Academic Medical Centre, Amsterdam, The Netherlands; 2 Bioinformatics Laboratory, Academic Medical Centre, Amsterdam, The Netherlands; 3 BiGCaT Bioinformatics, University of Maastricht, Maastricht, The Netherlands; 4 GeneGo, Inc., St. Joseph, MI, USA

## Abstract

**Background:**

The gut is a major energy consumer, but a comprehensive overview of the adaptive response to fasting is lacking. Gene-expression profiling, pathway analysis, and immunohistochemistry were therefore carried out on mouse small intestine after 0, 12, 24, and 72 hours of fasting.

**Results:**

Intestinal weight declined to 50% of control, but this loss of tissue mass was distributed proportionally among the gut's structural components, so that the microarrays' tissue base remained unaffected. Unsupervised hierarchical clustering of the microarrays revealed that the successive time points separated into distinct branches. Pathway analysis depicted a pronounced, but transient early response that peaked at 12 hours, and a late response that became progressively more pronounced with continued fasting. Early changes in gene expression were compatible with a cellular deficiency in glutamine, and metabolic adaptations directed at glutamine conservation, inhibition of pyruvate oxidation, stimulation of glutamate catabolism via aspartate and phosphoenolpyruvate to lactate, and enhanced fatty-acid oxidation and ketone-body synthesis. In addition, the expression of key genes involved in cell cycling and apoptosis was suppressed. At 24 hours of fasting, many of the early adaptive changes abated. Major changes upon continued fasting implied the production of glucose rather than lactate from carbohydrate backbones, a downregulation of fatty-acid oxidation and a very strong downregulation of the electron-transport chain. Cell cycling and apoptosis remained suppressed.

**Conclusion:**

The changes in gene expression indicate that the small intestine rapidly looses mass during fasting to generate lactate or glucose and ketone bodies. Meanwhile, intestinal architecture is maintained by downregulation of cell turnover.

## Background

In the postabsorptive state, the portal drained viscera (stomach, intestines, pancreas and spleen) and the liver account for 20–25% of the whole-body energy expenditure [1,2], even though these organs represent < 10% of body weight. The disproportional energy requirement of the gut is ascribed to the very rapid turnover of enterocytes and the continuous synthesis and degradation of mucous glycoproteins, which may serve to buffer amino-acid availability in the postabsorptive period [3,4]. A comprehensive view of the adaptive response of the intestine to maintain its integrity during food deprivation is, nevertheless, still lacking. Clinically, such insight is highly relevant to better understand the mucosal atrophy that develops as an undesirable sequel of parenteral nutrition [5]. Furthermore, not all functions of the gut may have been appreciated thus far. As an example, the long-term fasted gut was only recently shown to be capable of gluconeogenesis [6]. To obtain a more comprehensive understanding of the effects of short-term and prolonged food deprivation, we performed a microarray-based study of the effects of fasting in the mouse small intestine (SI). Although changes at the mRNA level cannot, of course, be directly extrapolated to metabolic adaptations, we show that the expression of genes involved in metabolism and cell turnover changed in a highly significant, coordinated manner, with a remarkably discontinuous transition between short-term and prolonged fasting. Most of the early responses to fasting were transient, peaking at 12 hours after food withdrawal, whereas the late response became more pronounced with the duration of fasting.

## Results

### Effects of fasting on intestinal structure

**Figure 1:**
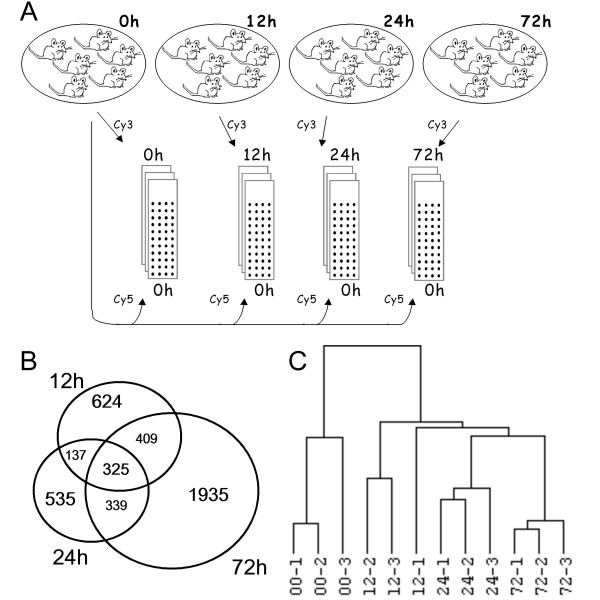
**Design of the microarray study**. A) Each of 3 microarrays per time point (0, 12, 24 and 72 hours of fasting) was hybridized with mRNA from a different pool of 2 animals, while mRNA obtained from a pool of 6 control intestines was used as reference. B) Numbers of significantly changed genes due to fasting: among 4304 genes differentially expressed (P < 0.01, ≥ 1.4-fold change), those changed at more than one time point of starvation are shown in the overlapping areas of the Venn diagram. C) Hierarchical clustering of microarrays shows segregation of different starvation time points into distinct branches of the dendrogram.

**Figure 2:**
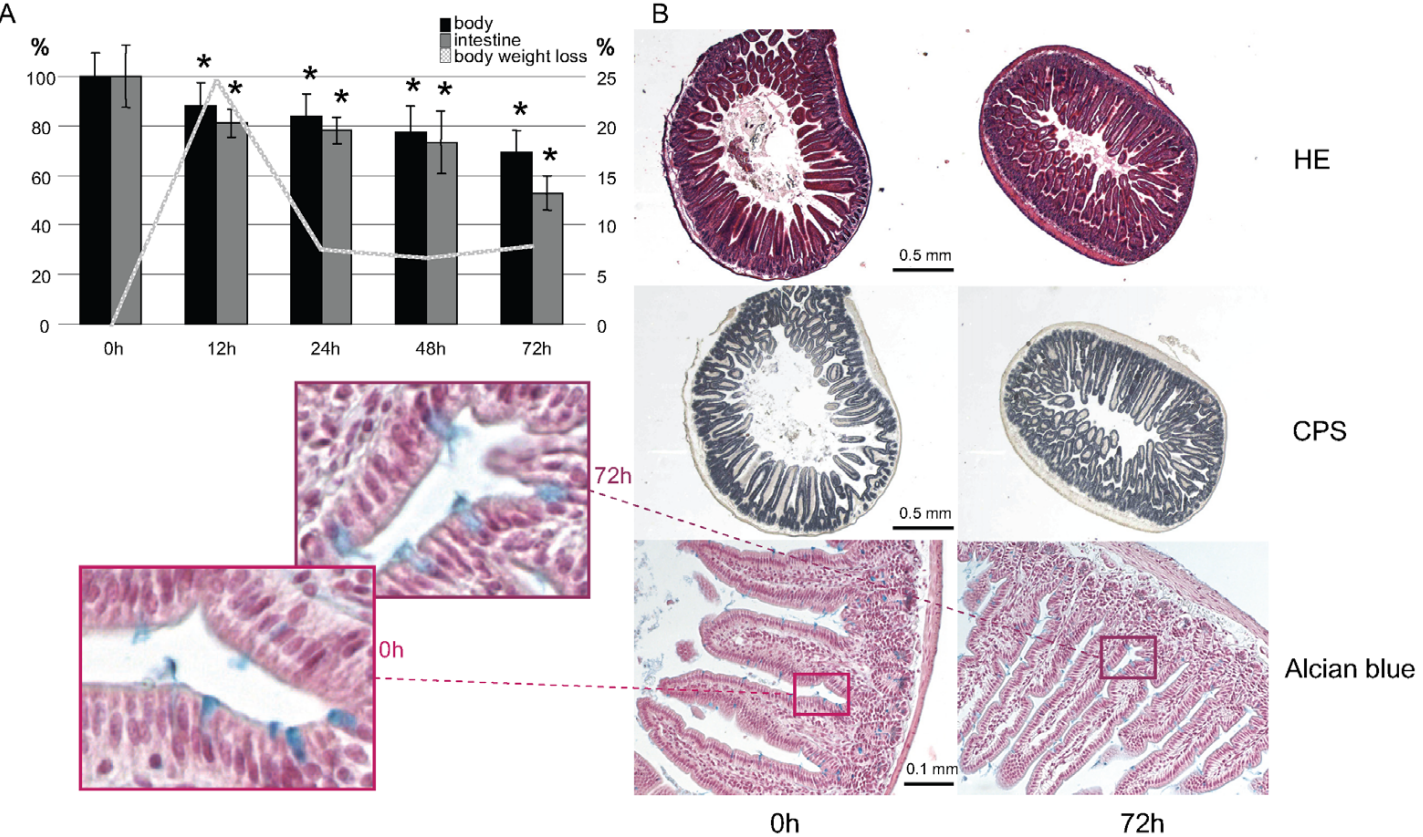
**Macro- and microscopic analysis of the fasting intestine**. A) Change in whole-body and intestinal weight during fasting as percentage of the control. Asterisks label significant changes in weight compared to the fed condition. The line represents the daily percentual change in body weight. B) Histology of representative intestinal samples at 0 and 72 hours of starvation (left and right panel, respectively) stained with hematoxylin and eosin (upper panels), carbamoylphosphate synthetase (middle panels), or Alcian blue (lower panels). Zoomed-in regions show blue-stained goblet cells. On the upper and middle panels bars represent 0.5, and on the lower Alcian blue-stained panel 0.1 mm.

**Figure 3:**
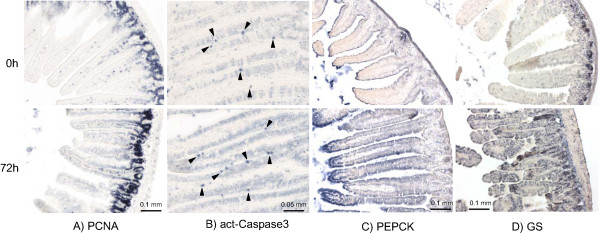
**Distribution of PCNA (A), active caspase 3 (B), phosphoenolpyruvate carboxykinase (C) and glutamine synthetase (D) protein **in fed and 72-hours fasted small intestine. The bars on the panels A, C and D represent 0.1 mm, and on B 0.05 mm.

To study the effect of fasting on the small intestine, 6 week old male FVB mice were subjected to fasting for 0, 12, 24 and 72 h and analyzed by means of immunohistochemistry and gene expression profiling (Figure 1A). During the first 12 hours of fasting, mice lost ~12% of their body weight (that is, 24% when expressed on a per-day basis). Thereafter, weight loss was steady at a rate of ~7% per day, so that mice had lost ~30% of their initial weight at 72 hours (Figure 2A). In Figure 2A the percentage of weight loss was used to give an insight into its cumulative reduction. Since the time intervals between the measurements were not identical, it was important to define a common denominator to determine the rate of body weight loss. For this reason, the rate of weight loss in consecutive time intervals was expressed as percent of weight loss per day. Gut wet weight declined more than body weight, having lost almost 50% of its initial weight after 72 hours of fasting. Small-intestinal weight loss was highest during the first 12 hours of fasting (~38% per day), low between 12 and 48 hours (~7% per day), to increase again between 48 and 72 hours (~29% per day). Protein content was only determined in fed and 48 h-fasted guts, declining approximately 20% in this period (Table 1). Changes in intestinal weight, therefore, reflect changes in intestinal protein content. Despite the pronounced loss of tissue mass, the basic morphology of the intestine remained unaffected (Figure 2B). In particular, the length of the villi did not change (Table 1). Using carbamoylphosphate synthetase (CPS, Figure 2B) as a marker for enterocytes and α-smooth-muscle actin (α-SMA, not shown) for smooth muscle, we could show that these two structural components accounted for ~75% and ~20%, respectively, of the intestinal volume in both fed and 72 h-fasted mice (Table 1). Alcian-blue staining for goblet cells showed no change in number between the fed and 72 h fasted condition (Table 1 and Figure 2B). These data demonstrate that fasting induces a proportional shrinkage of the components of the small intestine. The staining intensity of the DNA-synthesis marker PCNA increased 28% (P < 0.004) during the first 24 h of fasting and decreased to just 8% (P < 0.36) at 72 h (Figure 3A; Table 1), while the number of active caspase 3-positive cells (Figure 3B) had increased ~45% (24 h) and ~30% (72 h; Table 1) relative to the number before fasting. Since PCNA is both involved in DNA synthesis and repair, and since DNA synthesis is suppressed in the fasting intestine [7], these findings suggest that DNA repair mechanisms might be regulated.

**Table 1:** Body and organ weights and morphometrical measurements in fed and 72 h fasted mice

	fed	72 h fasting
body weight (g)	27.8 ± 0.9	18.9 ± 0.5*
intestinal wet weight (g)	1.2 ± 0.1	0.6 ± 0.1*
villus height (μm)	368 ± 63	353 ± 54
CPS-positive volume fraction (%)	72.2 ± 3.4	71.0 ± 0.5
αSMA-positive volume fraction (%)	24.6 ± 1.1	24.0 ± 3.0
PCNA volume fraction (%)	18.8 ± 1.4	20.6 ± 1.0
Alcian blue-positive cells^	68.7 ± 6.3	69.0 ± 11.1
caspase 3-positive cells^	56.0 ± 7.2	91.8 ± 14.9*
total protein content of the intestine^# ^(mg/g)	20.9 ± 2.2	16.8 ± 2.0*

Data are shown as average ± SEM. There are no significant differences between the experimental groups with regard to villus height, CPS-, αSMA- and PCNA-positive volume fraction, and number of Alcian-blue positive cells. Body and organ weight, caspase 3-positive cells, and total protein content differed significantly (*, P < 0.01). ^**: **for caspase 3- and Alcian blue-positive cells, the numbers are given per mm^2^. ^#^**: **total protein content of the gut was available for fed and 48 h-fasted animals only.

### Global gene-expression profile in the small intestine

Out of 7590 transcripts, including expressed sequence tags and RIKEN sequences, which met our boundary condition for significance (P < 0.01), 4304 (57%) were ≥ 1.4-fold up- or downregulated. Of these, 1495, 1336 and 3008 transcripts were ≥ 1.4-fold changed after 12, 24, and 72 hours of fasting, respectively (Figure 1B). [For a complete list of more than 1.4-fold up- or downregulated genes, see additional file 1.] A change higher than 2-fold occurred in 331, 196 and 903 genes after each of the fasting time points, respectively. A dendrogram (Figure 1C) generated by unsupervised hierarchical clustering of the arrays (using the entire probe collection), with correlation used as the similarity measure and average linkage as clustering parameters, revealed that the successive time points separated into distinct branches. The apparent predominance of biological over technical variation is a benchmark of the quality of the microarray analysis. Because the time points investigated represent different, successive phases of the fasting response, the set of transcripts that were uniquely up- or downregulated at each of the time points can be used as biological markers to follow the effects of interventions of the fasting regimen [see additional file 2, supplementary table 2].

### Quantitative real-time polymerase chain reaction analysis of selected transcripts

**Figure 4:**
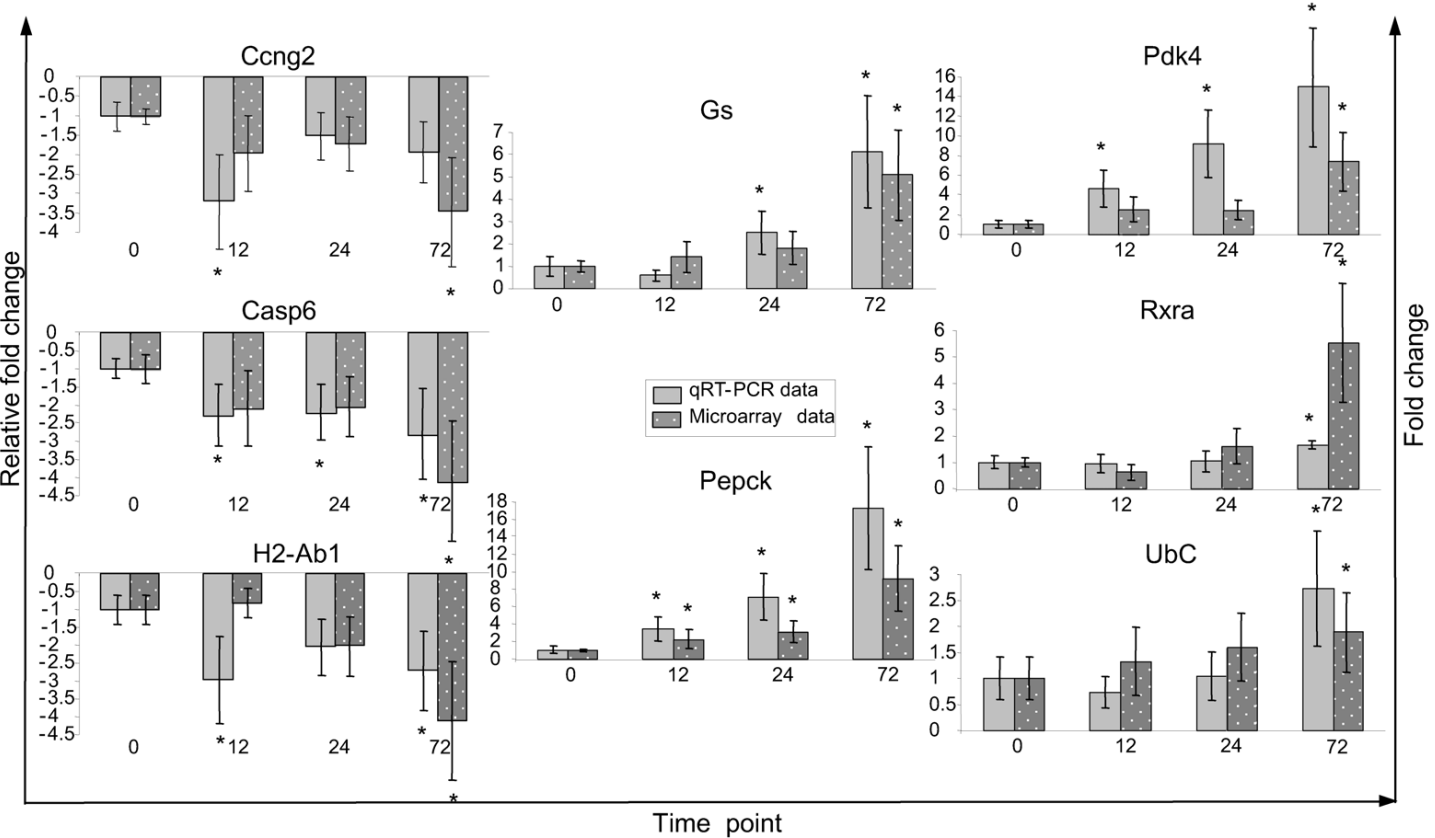
**Quantitative PCR analysis **of cyclin G2 (*Ccng2*), caspase 6 (*Casp6*), histocompatibility 2, class II antigen A, beta 1 (*H2-Ab1*), glutamine synthetase (*Gs*), phosphoenolpyruvate carboxykinase 1 (*Pepck1*), pyruvate dehydrogenase kinase 4 (*Pdk4*), retinoid × receptor α (*Rxrα*) and ubiquitin C (*UbC*) in duplicate samples of 6 mice. mRNA levels were related to 18S rRNA levels. Plain bars refer to microarray data, whereas spotted bars represent qPCR data. Asterisks indicate significant change in comparison to 0 h (P < 0.05; n = 6).

The quantitative nature of the microarray data was validated with qPCR, using 18S ribosomal RNA as an internal reference. Of the 8 genes studied, 5 were up- and 3 downregulated by fasting, responding with a 1.3–9.2-fold change in expression on the microarrays. Irrespective of whether the transcripts were rare (*Gs *and *Pdk4*) or abundant (*Pepck *and *UbC*), their changes in expression were similar in the microarray and qPCR quantifications (Figure 4). The increase in *Gs *and *Pepck *expression was of interest, since the number of enterocytes expressing these enzymes increased concomitantly (Figure 3). The main difference between the time points is much more intense staining at the cytoplasm of the enterocytes, while the nuclei remained unstained.

### Global analysis reveals a transient early and gradual late intestinal response to fasting

**Figure 5:**
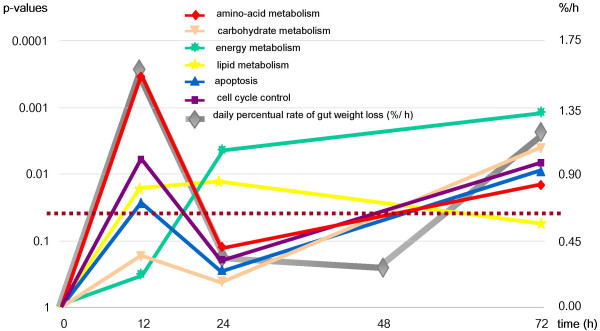
**Adaptive changes in metabolic and cellular processes in the small intestine during fasting**. The significance of changes in mRNA levels within (a group of) pathways across time was analysed with MetaCore™ software. The P-values in the pathways are calculated using the hypergeometric distribution, where the P-value represents the probability of particular mapping arising by chance, given the numbers of genes in the set of all genes in pathways, genes in a particular pathway and genes in the present experiment. The pathways are grouped into processes as defined in MetaCore™ version 3.0. The dotted line represents the significance threshold (0.05). The daily percentual change of intestinal weight during fasting is shown on the secondary y-axis.

Cluster analysis did not reveal biologically meaningful groups of genes. In this respect, GenMAPP and, in particular, MetaCore™ were more informative tools to search for coordinate changes in metabolic pathways. The biochemical and signaling pathways in the small intestine that were affected by fasting were identified using P < 0.01 and ≥ 1.4-fold change as thresholds for individual genes. Figure 5, based on the MetaCore™ approach, reveals that pathways involved in amino-acid, energy, lipid, and carbohydrate metabolism, apoptosis, and cell-cycle control responded with a significant change in expression upon fasting. The graph in Figure 5 presents P-values as parameter of the likelihood that coordinate changes in the pathways shown were indeed present at the different time points of fasting. As statistical parameter, the P-value encompasses no variation. The changes in amino-acid metabolism, cell-cycle and apoptosis were remarkably biphasic, with a transient early response that peaked at 12 hours after food withdrawal and a late response that became more pronounced with the duration of fasting. Since all animals were sacrificed at the same time point during the day, the early, transient effects of fasting cannot be ascribed to a circadian rhythm. The figure further reveals that the changes in energy and carbohydrate metabolism became more pronounced with continued fasting, whereas lipid catabolism was no longer regulated at 72 hours. The common denominator of the transient early response appeared to be amino-acid metabolism and cell turnover (cell cycle, apoptosis), whereas the late response was associated with energy metabolism (carbohydrate and energy metabolism) and cell turnover. However, this global analysis does not reveal the direction of the changes and lacks functional detail. In the next sections, we therefore scrutinize some of the individual pathways to deduce the putative functional consequences.

### Fatty-acid catabolism

**Figure 6:**
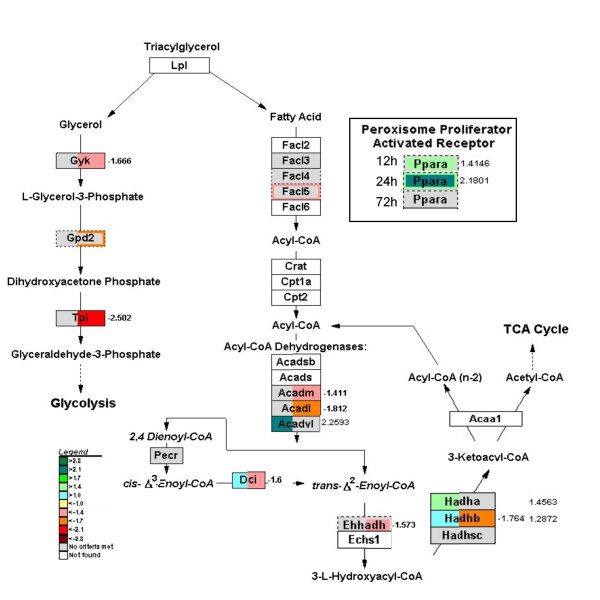
**GenMAPP pathway showing changes in expression of the transcription factor PPARα and fatty-acid degrading enzymes upon fasting**. The upregulation of *Pparα *correlates with the upregulation of fatty acid β-oxidation involved *Acadvl *and *Hadha *at 12 h of fasting, while the prolonged fasting provokes downregulation of 6 genes involved in this process and return to control values of *Pparα *expression (regulation of PPARα target genes involved in other processes is shown in the additional file 2, supplementary table 3). Warm colors (from yellow to red) represent down-regulation, while cold (light blue to dark green) indicate an induction (with exact fold-change shown aside the boxes). Gray indicates no significant change. Genes not coupled to reporters on the array are shown in white. Genes represented by more than one sequence on the array are shown in a dash-lined box with the level of change depicted by a colored line surrounding the field.

**Figure 7:**
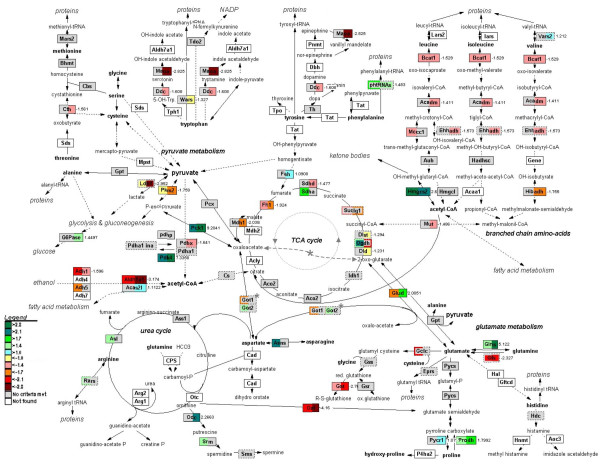
**Changes in expression of amino-acid metabolizing enzymes in the fasting mouse intestine**. Changes observed after 12 or 72 hours of starvation are shown in color code on the left and right side of the enzyme box, respectively. The color code is the same as in Figure 6. The asterisk indicates the position of Got2.

**Figure 8:**
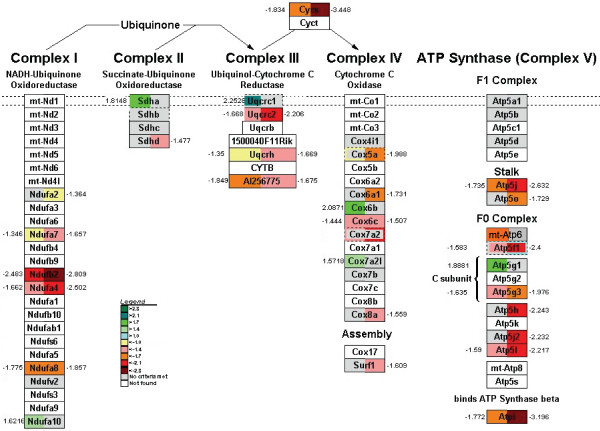
**GenMAPP showing changes in expression of the electron-transport chain during fasting**. At 12 hours of fasting, 14 out of 39 respiratory chain genes linked to our data were downregulated, whereas 6 genes, scattered over all 5 complexes, were upregulated. At 72 hours, 4 out of 7 complex-I genes, 1 out of 4 complex-II genes, 3 out of 4 complex-III genes, 6 out of 10 complex-IV genes, 8 out of 13 complex-V genes, and cytochrome C were downregulated. The color code and map description are the same as in Figure 6.

**Figure 9:**
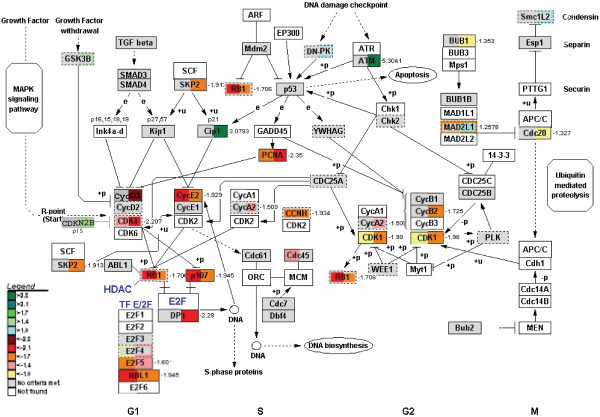
**GenMAPP showing changes in expression of cell-cycle regulating genes upon fasting**. Nine cyclin- and cyclin-dependent kinase-coding genes show moderate to strong downregulation at both early and prolonged fasting, while 3 of their inhibitors are upregulated in prolonged starvation. At least 4 regulators of cell-cycle transition are also strongly downregulated, indicating overall slow-down of the cell turnover. For the color code and map description see Figure 6.

**Figure 10:**
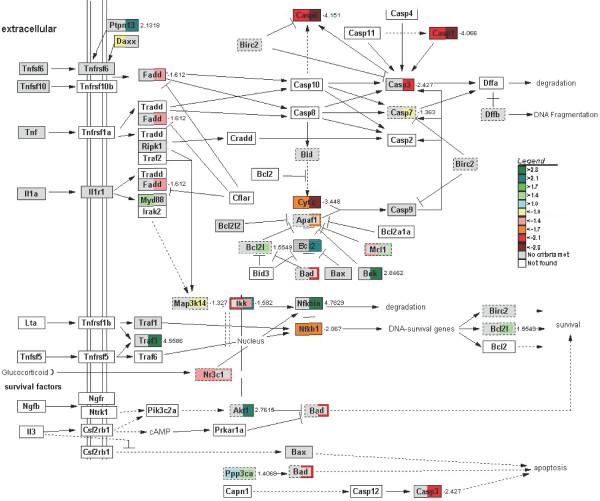
**GenMAPP showing changes in expression of apoptosis regulating genes upon fasting**. Proapoptotic genes are shown to be down-, and antiapoptotic upregulated in both early and extended fasting. The color code and map description are the same as in Figure 6.

The expression of the transcription factor *Pparα*, a major regulator of fatty-acid oxidation, was upregulated at 12 hours and even more so at 24 hours of fasting. The genes that are involved in the first stage of very long-chain fatty-acid oxidation (acyl-coenzyme A dehydrogenase (*Acadyl*) and the α-subunit of the trifunctional protein (*Hadha*)) were upregulated at 12 hours of fasting, but this increase changed to down-regulation at 72 hours (Figure 6). [For a complete list of *Pparα *– regulated genes see additional file 2, supplementary table 3; the gene lists that are specific for pathways in Figures 6, 7, 8, 9, 10 are shown in additional file 3.] The expression of HMGCoA synthase2 was stimulated throughout the fasting period (Figure 7), facilitating the synthesis of ketone bodies from acetyl-CoA. 3-Hydroxy-3-methylglutaryl-coenzyme A reductase (*Hmgcr*), the rate-determining enzyme of sterol biosynthesis, was downregulated 1.5-2-fold at all three time points (data not shown). Finally, genes involved in glycerol metabolism (glycerol kinase (*Gyk*), mitochondrial glycerolphosphate dehydrogenase 2 (*Gpd2*) and triosephosphate isomerase (*Tpi*)) became downregulated during prolonged fasting, whereas the fatty-acid transporter *Cd36 *became upregulated (not shown). These data underscore that lipid catabolism subserves energy metabolism in the small intestine during the first day of fasting only.

### Amino-acid and carbohydrate-backbone metabolism

Of all pathways studied in the fasting gut, the adaptive changes in amino-acid metabolism were most pronounced, showing a predominant but transient response on the first day of fasting and another, less pronounced response during the later phase (Figure 5). To generate an overall view of these adaptive changes, we created a cumulative map of amino-acid metabolizing pathways within the GenMAPP environment (Figure 7).

#### Early changes

Most of the early changes in amino-acid metabolism converge on glutamine and glutamate. The 2.5-fold increased concentration of asparagine synthetase (*Asns*) mRNA is indicative for a deficiency in cellular amino acids, in particular glutamine [8]. The downregulation of cytosolic glutamate-consuming γ-glutamylcysteine synthetase (*Gclc*) and the upregulation of glutamine synthetase can therefore be interpreted as an effort to preserve the cytosolic glutamine levels. The strong reduction in expression of mitochondrial glutaminase (*Gls*) points to preservation of mitochondrial glutamine and reduction of ammonia production. Similarly, the strongly decreased expression of mitochondrial ornithine transaminase (*Oat*), which, in enterocytes, transaminates glutamate to ornithine [9], and the increased expression of mitochondrial proline oxidase (/dehydrogenase – *Prodh*) both have a sparing effect on mitochondrial glutamate. Furthermore, the 2-fold downregulation of mitochondrial glutamate dehydrogenase (*Glud*), in conjunction with the enhanced NADH production during fatty acid oxidation, suggests that mitochondrial glutamate does not feed the citric-acid cycle via deamination. Finally, mitochondrial branched-chain amino-acid transaminase (*Bcat1*) was downregulated.

The citric-acid cycle becomes supplied with acetyl-CoA from fatty-acid oxidation rather than from pyruvate, as the 2.5-fold upregulation of *Pdk4 *expression almost certainly inactivates pyruvate dehydrogenase. Although expression of pyruvate carboxylase (*Pkm2*) was not changed, its activity is enhanced by acetyl-CoA. The upregulation of α-oxoglutarate dehydrogenase (*Ogdh*) and succinate dehydrogenase (*Sdha*) further suggests that mitochondrial ATP synthesis is initially spared. The combination of the downregulation of enzymes consuming mitochondrial glutamate (see previous paragraph) and the upregulation of mitochondrial glutamate-oxaloacetate transaminase (*Got2*) suggests enhanced cycling through a truncated portion of the citric-acid cycle [10]. The very pronounced upregulation of *Pepck *expression points towards a stimulation of the consumption of oxaloacetate for PEP synthesis and, since glucose-6-phosphatase is not upregulated, lactate production. In aggregate, the data point to changes favoring the conservation of glutamine, an inhibition of pyruvate oxidation, a preserved capacity for oxaloacetate synthesis, and an enhanced capacity for PEP synthesis. The demand for oxaloacetate, in conjunction with the 3.8-fold upregulation of *Hmgcs2*, indicates that acetyl-CoA from fatty-acid oxidation also stimulates the synthesis of ketone bodies.

#### Prolonged fasting

At 72 hours of fasting (Figure 7), mitochondrial glutaminase was still strongly inhibited, whereas *Gs *expression was still very strongly upregulated. Mitochondrial glutamate metabolism still appeared to be conserved for α-ketoglutarate production (ornithine aminotransferase expression was very strongly suppressed, whereas proline oxidase was stimulated 2-fold), but *Gldh *expression was 2-fold stimulated and *Got2 *expression had returned to fed values. Because NADH production from fatty-acid oxidation was no longer stimulated, these changes indicate that glutamate now feeds the citric-acid cycle via deamination. *Pdk4 *and *Hmgcs2 *were still strongly upregulated, suggesting that pyruvate oxidation was still inhibited and ketone-body synthesis stimulated. *Pepck *expression was still strongly upregulated. In addition, the expression of glucose-6-phosphatase (*G6Pase*) increased 1.5-fold, whereas that of lactate dehydrogenase had decreased almost 3-fold, suggesting that the enterocytes had acquired the capacity to produce glucose rather than lactate from amino acids, in particular glutamate. Several steps in BCAA and tryptophan metabolism were strongly downregulated, probably to conserve these essential amino acids.

The 2-fold downregulation of glucosamine 6-phosphate N-acetyltransferase across the entire fasting period, together with more than 2-fold upregulation of glucosamine-fructose-6-phosphate aminotransferase at 24 hours, suggest an inhibition of UDP-GlcNAc synthesis and a decline of O-glycosylation. An obvious target is mucus production.

Alcohol-metabolizing enzymes (alcohol dehydrogenases 1 and 5 (*Adh1 *and *-5*) and aldehyde dehydrogenase 1a1 (*Aldh1a1*; Figure 7), involved in the oxidation and elimination of metabolic alcohols and aldehydes, including products of lipid peroxidation [11], were downregulated.

### Electron-transport chain

The genes of the electron-transport chain responded progressively to fasting. At 12 hours of fasting, 14 genes (out of 39 linked to our data) were downregulated, whereas 6 genes, scattered over all 5 complexes, were upregulated (Figure 8). At 24 hours, only 3 genes remained upregulated among 20 downregulated species. At 72 hours of fasting, no fewer than 62% of the respiratory chain genes, including cytochrome C, were downregulated (for details, see Figure 8). Taken together, these data indicate that the capacity for ATP synthesis becomes strongly suppressed in the empty gut.

### Cell cycle and apoptosis in response to nutrient deprivation

Figure 9 shows that many genes involved in cell-cycle regulation, in particular the cyclins (*Cyca2*, *Cycb2*, *Cycd*, *Cyce1*, *Cych*), became downregulated in the course of fasting. In addition, a number of cyclin-dependent kinases (*Cdk1*, *Cdk4*), proliferating-cell nuclear antigen (*Pcna*), and regulators of genes that act within the cell cycle, like retinoblastoma (*Rb1*) and retinoblastoma-like protein (*Rbl1*), were downregulated, whereas inhibitors of cyclin-dependent kinases (CDK inhibitor 1 (*p21*, *Cip1*); CDK4 inhibitor (*p15*, *Cdkn2b*)) were upregulated. Insulin-induced protein-1 (*Insig-1*, not shown), which plays a regulatory role during the G0/G1 transition, was also strongly down-regulated (2.4-3-fold). The strong upregulation of the cell-cycle inhibitors *Atm1 *(which phosphorylates among others p53 and NFκBIA) and glycogen synthase kinase 3β (*Gsk3b*) fits within the picture of a slowdown of the cell cycle. The oxidative stress associated with the downregulation of γ-glutamylcysteine synthetase (Figure 7) would also inhibit cell proliferation [12].

The finding that PCNA protein as visualized with immunostaining, was more prominently present at 24 h of fasting (Figure 3), demonstrates that the observed changes in mRNA levels (-2.1, -1.7 and -2.4 fold respectively) reveal activity of signaling routes, which become reflected in protein levels with a delay. Among other genes involved in DNA repair, the critical serine-threonine kinase ATM, which regulates the checkpoint signaling due to the double-strand breaks, was upregulated (1.9 and 5.3 fold at 24 and 72 h, respectively). The 'executive' genes, however, like DNA polymeraseβ, nibrin, or double strand-break repair-protein MRE11, were all downregulated at one or more time points (data not shown). It is therefore difficult to draw a straightforward conclusion on the overall effect of fasting on DNA repair.

In addition to the expression of genes involved in cell proliferation, the expression of genes involved in apoptosis was suppressed. We modified the apoptosis map in the GenMAPP environment to visualize this adaptive response (Figure 10). The caspase-cascade elements *Casp1 *and *6*, and cytochrome C became downregulated early during fasting, the effect being most pronounced at 3 days of fasting. The expression of the apoptosis antagonist *Bcl2 *and *Bcl2l *(*Bcl-xl*), which prevent the release of cytochrome C from mitochondria, and the pro-survival *Akt *became strongly upregulated at 72 hours, whereas the Bcl-associated death promoter (*Bad*), apoptotic protease-activating factor 1 (*Apaf1*), and caspases 1, 3 and 6 were strongly down-regulated. The notion that cell turnover is suppressed is underscored by the relatively mild increase in number of active caspase 3-positive cells (~30%) after 3 days of fasting.

Autophagy is another well-known adaptive response to nutrient starvation [13]. We did not notice a major change in genes involved in autophagy, but only few were represented on the arrays.

## Discussion

### Phases in the adaptive response to fasting

Based on the rate of weight loss after food withdrawal, the body passes through 3 successive adaptive phases ([14]; Figure 2). During the first, postabsorptive fasting phase, the rate of weight loss is relatively high (~24% per day in mice (Figure 2) and ~10% per day in rats [15,16]). During the intermediate, "coping" phase, the loss of body mass is slower (~7% per day in mice and ~6% per day in rats [15]). During the last, preterminal fasting phase, the loss of body weight again increases (~9% in rats [15]). We did not observe a preterminal phase with an increased rate of body-weight loss in our mice. At 72 h, our mice still looked vital. One explanation could therefore be that they had not yet or had just entered the preterminal phase of body-weight loss. Alternatively, mice, unlike rats, do not have the preterminal phase characterized by an increased weight loss. The initial phase of fasting is accompanied, at the whole body level, by a decline in circulating insulin, glucose, triglycerides and cholesterol levels [16], a reduction in protein synthesis and an increase in protein degradation, while the intermediate, coping phase shows a temporary increase in circulating ketone bodies [17] and free fatty acids [18], and a decline in protein degradation [14]. The final, preterminal phase of fasting is accompanied by an increase in circulating corticosterone and plasma urea levels and a further decline in whole-body protein synthesis [15,17,19]. From these data, it was concluded that mammals adapt to prolonged fasting by mobilizing fat stores and minimizing protein loss. This model was then implicitly expanded to all organs separately, including the gut.

However, microarray studies that have prospected the adaptive response to fasting of the small intestine (present study), liver [20], and muscle [21-23], and more limited studies in kidney [24], reveal a different scenario. Liver, muscle, and kidney respond to fasting with a progressive change over time in mRNA concentrations of enzymes involved in protein, carbohydrate and fat metabolism. The small intestine differs from these organs in its biphasic response to fasting, that is, the small intestine mounts, in addition to the slow, progressive changes in expression that are also seen in liver, muscle, and kidney, a pronounced, but transient early response. In this early phase of fasting, gene expression changes in a direction that facilitates the preservation of glutamine, the catabolism of glutamate to ATP and lactate, and the suppression of excessive cell turnover. The progressive late response is numerically characterized by a larger number of affected genes (Figure 1B), higher fold changes in expression (not shown), and functionally by changes in gene expression that favor, in addition to suppression of cell turnover, glucose rather than lactate production.

### Adaptive changes in intestinal morphology and cell turnover

Our data show that weight loss in response to fasting is more pronounced in the small intestine than in the body at large. The relative loss of intestinal wet weight upon fasting is similar in mice (Figures 2 and 5) and rats [7,25]. Mice differ from rats in that villar height, crypt depth, and cell number per villus or crypt are not (Figure 2 and [26]), or hardly [27] affected by fasting, whereas in fasting rats crypt and villar length decline to ~60% [7,25]. Apart from villar density, no changes in the relative volume of the mucosa (measured as CPS-positive volume fraction), of goblet cells (measured as the number of Alcian blue-positive cells), and smooth muscle (measured as αSMA-positive volume fraction) develop in fasting mice (Figure 2 and Table 1). The important consequence of these findings is that fasting induces a proportional shrinkage of all components of the small intestine in mice, that is, the contribution of different cell types remains unchanged. The tissue base for the microarray data does, therefore, not change in the course of the experiment.

Mechanisms that may account for the preservation of intestinal architecture are the suppression of the normally high cellular turnover, that is, cell multiplication [26,17] and apoptosis [26]. In both rats [28] and mice (Figure 3, Table 1), starvation is accompanied by an increase in the number of apoptotic cells in the epithelium, but in view of the simultaneously occurring proportional reduction of all structural components, the susceptibility to undergo apoptosis must be decreased in the surviving cells [29]. Accordingly, the microarray data reveal a regulation of the *Bcl2 *gene family. In agreement with their longer survival, the enterocytes of fasting intestines look more mature that those in the fed intestine [30,31]. The spreading of the expression of PEPCK and GS across the entire villus is also compatible with a more differentiated phenotype of the enterocytes. The microarrays also indicate a downregulation of cell-cycle genes. This is in apparent contrast to the elevated PCNA staining. Most studies show inhibition of cell proliferation during fasting [7,25], but an increased DNA synthesis in the rat intestine has been reported upon prolonged fasting [25]. We interpret these observations as indicating that DNA repair rather than cell proliferation is stimulated to maintain epithelial integrity.

### Adaptive changes in intestinal metabolism – early adjustments

**Figure 11:**
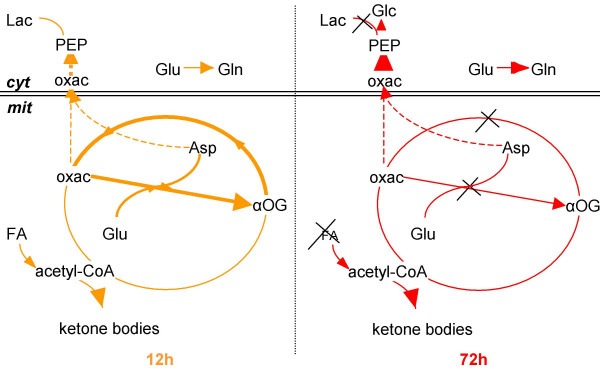
**A schematic model of the response to fasting in the mouse small intestine**. The adaptations of protein, fat and intermediary metabolism in the small intestine are indicated. Arrows and crosses represent up- and downregulation of the process respectively, in short (12 h, left) and prolonged fasting (72 h, right). Abbreviations for cytosolic (cyt) and mitochondrial (mit) metabolites stand for: Asp – aspartate, FA – fatty acids, Glc – glucose, Gln – glutamine, Glu – glutamate, Lac – lactate, αOG – α oxo-glutarate, oxac – oxaloacetate, and PEP – phosphoenolpyruvate.

The MetaCore analysis revealed that the adaptive changes in intestinal metabolism converge, as could perhaps be expected, on energy metabolism (Figure 11). The upregulation of asparagine synthetase was an informative starting point, as it revealed that intracellular glutamine levels were limiting [8]. Since the landmark experiments of Windmueller and Spaeth 25–30 years ago [32], it is known that the small intestine of rodents is an avid consumer of glutamine in addition to glucose for its energy supply. In agreement, the observed changes in mRNA levels of amino-acid metabolizing enzymes were concentrated in the glutamate family of amino acids (glutamine, glutamate, proline, arginine [33]). Short-term fasting is typically associated with an increased concentration of free fatty acids in the circulation [34]. Intracellular oxidation of fatty acids causes an increase in the intramitochondrial acetylCoA/CoA and NADH/NAD^+ ^ratios and an inhibition of pyruvate dehydrogenase [10]. In agreement, our data pointed to a strong inhibition of pyruvate oxidation, as well as an enhanced synthesis of ketone bodies during fatty-acid oxidation (while the high NADH/NAD^+ ^ratio inhibits deamination of glutamate). The data also suggested an enhanced cycling in a truncated citric-acid cycle from glutamate via α-ketoglutarate and succinate to oxaloacetate and back to glutamate. This truncated citric-acid cycle appeared to be fed by glutamate, because mitochondrial enzymes catabolizing glutamate were downregulated. In addition to glutamate, a number of amino acids and odd-chain fatty acids could feed this minicycle and yield ATP. The high intramitochondrial NADH/NAD^+ ^ratio, which facilitates malate formation from oxaloacetate and export to the cytosol, together with the very strong upregulation of *Pepck*, suggested enhanced PEP synthesis. In view of the very low *G6Pase *expression, PEP is probably converted to pyruvate, again yielding an ATP, and then lactate as substrate for gluconeogenesis in the liver [35-37]. It is well possible that BCAA catabolism is inhibited to avoid excessive drainage of these essential amino acids.

### The role of PPARa in the early response to fasting

The prominent role of peroxisome proliferator-activated receptor α (PPARα) in the adaptive response to fasting is well documented [38]. In agreement, we observed that *Pparα *itself and a number of PPARα-dependent genes involved in fatty-acid β-oxidation (e.g. *Acadvl*, *Dci*) and ketone-body synthesis (e.g. *Hmgcs2*) were induced in the intestine upon fasting. Ketone-body production is a well-known response to fasting in the liver [32,39], but not in the adult intestine. However, the suckling intestine does produce ketone bodies until weaning [40]. Our data indicate that the capacity to produce ketone bodies is reactivated upon short-term fasting. Production of ketone bodies in the gut may contribute to the increased ketonemia in prolonged fasting [39].

In addition to genes involved in fatty-acid oxidation, many other PPARα-target genes shown in supplementary Table 3 (e.g. *Cte1*, *Fabp4 *and *ScdI*) exhibited the early, transient induction pattern. Furthermore, PPARα regulates the expression of caspase 3 and amino-acid metabolizing enzymes, also in the gut [41,42]. In aggregate, our findings therefore extend the earlier finding that PPARα functions as a dominant regulatory factor in the response to fasting of the gut.

### The late response to fasting

Among the major changes that characterize the late response to fasting are the downregulation of fatty-acid oxidation, the severe downregulation of genes involved in the respiratory chain, and the upregulation of glucose synthesis (Figure 11). Whether or not the fasting gut produces glucose has attracted much interest lately [36,43]. We did observe that the expression of *Pepck *and *G6Pase *mRNA is increased, while that of *Ldh1 *is strongly downregulated. Metabolic studies indicate that the small intestine may account for 30–35% of total body gluconeogenesis after prolonged fasting [36,43]. Although we studied only changes in mRNA levels, we did also observe that glycogen, which completely disappears from murine liver within 12 hours of fasting, accumulates to a high level in the pericentral zone of the liver lobule after 72 hours of fasting (Sokolovic M, unpublished data). The source of pericentral glycogen is blood glucose [44], whereas amino acids and lactate typically generate periportal glycogen [45]. However, the generation of ATP necessary for gut gluconeogenesis must become increasingly precarious, because the respiratory chain becomes progressively inhibited (Figure 8).

### Limitations of the study

It goes without saying that our interpretations have their caveats if extrapolated to larger mammals. The mouse, as a very small animal, survives at most 4 days without food [46], whereas rats survive more than 2 weeks [47] and humans 2 months [48]. The degree to which intestinal mucosa is affected by fasting appears to vary between species. Rat and pig have been reported to lose up to 40 and 35 % of intestinal weight as a result of fasting, respectively [7,25,49]. The villar height decreases up to 50% in fasted rats and 30% in fasted piglets. Only a modest mucosal atrophy (i.e. = 10 % decrease in thickness) was found in critically ill humans [5]. Our findings on preserved gut morphology in the mouse, therefore, resemble the situation in humans.

Another obvious caveat of the present study is that only adaptive changes in cellular mRNA concentration were analyzed and used to construct the adaptive metabolic response. Most adaptive changes occur more rapidly at the mRNA than at the protein level. Furthermore, not all changes at the mRNA level perspire to changes at the protein level, whereas more than 500 known posttranslational modifications [50] do not need changes in mRNA level to affect the activity of proteins. The changes at the mRNA level that we report do probably reflect changes in the signal-transduction network that mediates the adaptive changes in metabolism and cell turnover better than that they reflect the changes in protein levels. The changes in signal transduction that modulate metabolite flow, the cell cycle and apoptosis are presently being analyzed.

## Conclusion

Our study shows that the adaptive changes in gene expression in the murine small intestine that are induced by fasting are directed at the induction of a proportional shrinkage of the tissue components of the small intestine. The preservation of the overall architecture of the organ is achieved by converting proteins and fats into energy substrates, while suppressing excess cell turnover. The response to fasting is biphasic, with an early, postabsorptive response that peaks at 12 hours and a late response that becomes more pronounced with continued fasting. The two phases differ in that gene expression during the early adaptive phase changes in a direction that facilitated the preservation of glutamine, the catabolism of glutamate to lactate, and the catabolism of fats to ketone bodies. Prolonged fasting appeared to induce the production of glucose rather than lactate from carbohydrate backbones, a downregulation of fatty-acid oxidation and a very strong downregulation of the electron-transport chain.

## Methods

### Animals

Male FVB mice (Charles River, Maastricht, The Netherlands) were housed at 20–22°C, 50–60% humidity, a 12 hours light/dark cycle, and food and water ad libitum. At 6 weeks of age, mice were fasted by removing chow for up to 72 hours before sacrifice (n ≥ 8 per group). The animals were kept in metabolic cages to prevent the consumption of beddings and were kept warm with an infrared lamp starting at 24 h. The daily rate of body or organ mass loss was calculated as described [29]. The study followed the Dutch guidelines for the use of experimental animals and was approved by the AMC Animal Experiments Committee.

### Tissues

All animals were sacrificed between 9:00 and 10:00 a.m. by cervical dislocation. The small intestine was removed quickly in such a way that adherent tissue remained behind. A central, 1 cm-long fragment was divided in two and fixed overnight at 4°C in 4% buffered formaldehyde or a mixture of methanol, acetone, and water (2:2:1 by volume). The remaining parts of the SI were opened longitudinally, rinsed in phosphate-buffered saline, blotted, weighed, snap-frozen in liquid N_2_, and stored at -80°C. We opted to use extracts of full-thickness intestine for gene-expression profiling, because the epithelial component of the murine small intestine comprises over 70% of its volume (see Figure 2). The suitability of this strategy is underscored by a recent microarray study of transporters in the mouse intestine [51]. In addition, isolation of enterocytes [52,53] is time-consuming and, hence, entails a risk of mRNA degradation, while mucosal scraping harvests villi more efficiently than crypts [54], whereas many mRNAs are most abundant in the crypts.

### RNA isolation and quantification

Total intestinal RNA was extracted from frozen tissue with guanidiniumthiocyanate [55], followed by cesium-chloride centrifugation [56] to avoid contamination with mucus. The quality of RNA was assessed with the RNA 6000 Nano LabChip^® ^Kit in an Agilent 2100 bioanalyzer (Agilent Technologies, Palo Alto, USA). Given the high sensitivity of Agilent arrays [57], we opted for 1.4-fold change as inclusion criterion for a gene. Microarray-based quantification of 8 mRNAs with a 1.3–9.2-fold change in expression was validated by qPCR, as described [58]. mRNA concentration was calculated using the LinReg program [59]. In the absence of reverse transcriptase, the signal was < 0.1% of that in its presence for each primer pair (not shown). Gene-specific primer sequences, product lengths, annealing temperatures and MgCl_2 _concentrations are shown in supplementary table 1 [see additional file 2].

### Microarrays

The 60-mer Mouse Development (22 K) Oligo Microarray G4120A (Agilent) was used. Three arrays per experimental condition were used. Per microarray, 20 μg mRNA, pooled from 2 intestines, was reverse transcribed with Cy3-labelled dCTP (Perkin Elmer, Boston, USA), using the Agilent Fluorescent Direct Label Kit. Cy5-labeled cDNA produced from RNA pooled from 6 fed animals served as the common reference across all arrays (Figure 1A). Hybridized cDNAs were detected with Agilent's dual-laser microarray slide scanner. The data were retrieved with Agilent's Feature Extraction software 6.1.1.

### Data analysis

We opted for the reference design, because it is robust to bad arrays, amenable to clustering and, more importantly, allows for comparison of more than 2 classes (time points) at once, without dye effects [60]. Foreground and background median signals were used to calculate background-subtracted intensities. The resulting data were normalized with the quantile normalization method as a preparatory step for the application of the Split-Factor ANOVA. Quantile normalization equalizes the distribution on arrays and thus limits the influence of technical errors [61,62]. Outliers were removed with a separate ANOVA model, specially developed to detect local artifacts due to scratches, blurs, dust, etc. within the common reference channel [63]. This procedure also detects non-uniform hybridization. Genes differing significantly in expression in the respective experimental conditions were identified by applying a Split-Factor ANOVA model to both the green (experimental) vs. red (reference) measurements, and the indirect, across-array comparison of the Cy3 measurements of starved vs. fed animals. The split-factor ANOVA divides the entire set of test samples into individual samples, which allows a sensitive detection of differentially expressed genes. A comparison with both the reference samples and the other test samples within the same set provides an additional test for the presence of any remaining false positives due to non-eliminated outliers. A consensus between the direct and across-array ANOVA ensures that final results do not suffer from either dye-gene effects or array-specific noise. Only genes that received a significance call in 2 out of 3 microarrays in both the direct and across-array split-factor ANOVA were taken into further consideration. In all applications, P < 0.01 and = 1.4-fold change were used as gene-inclusion criteria.

Cluster analysis was performed using the publicly available programs "Cluster" [64,65] and "TreeView" [66,67].

Pathway analysis and visualization was carried out using GenMAPP [68] (2.0 β-version, using the Mm-Std_20040411 gene database and 20040426_Mm version of local maps) and MappFinder [69] (Gladstone Institutes, UCSF, San Francisco, USA) software. GenMAPP was also used to build the map shown in Figure 7 and to modify those in Figures 6 and 10. The complexity of the response required a system-wide approach to data analysis, so the MetaCore™ software (GeneGo, Inc., St. Joseph, MI, USA) was used to assess the significance of changes in expression of genes in specified pathways [70,71]. Significance of changes in expression in pathways or networks is evaluated in the MetaCore™ suite based on the size of the overlap between user's dataset and a set of genes corresponding to a network or pathway queried. This problem is cast as the probability that randomly obtained overlap of certain size between the user's set and a network/pathway follows a hypergeometric distribution:

P(r,n,R,N)=CRr⋅CN−Rn−rCNn=Cnr⋅CN−nR−rCNR=R!(N−R)!N!⋅n!(N−n)!r!(R−r)!⋅1(n−r)!(N−R−n+r)!
 MathType@MTEF@5@5@+=feaafiart1ev1aaatCvAUfKttLearuWrP9MDH5MBPbIqV92AaeXatLxBI9gBaebbnrfifHhDYfgasaacH8akY=wiFfYdH8Gipec8Eeeu0xXdbba9frFj0=OqFfea0dXdd9vqai=hGuQ8kuc9pgc9s8qqaq=dirpe0xb9q8qiLsFr0=vr0=vr0dc8meaabaqaciaacaGaaeqabaqabeGadaaakeaacqWGqbaucqGGOaakcqWGYbGCcqGGSaalcqWGUbGBcqGGSaalcqWGsbGucqGGSaalcqWGobGtcqGGPaqkcqGH9aqpdaWcaaqaaiabdoeadnaaDaaaleaacqWGsbGuaeaacqWGYbGCaaGccqGHflY1cqWGdbWqdaqhaaWcbaGaemOta4KaeyOeI0IaemOuaifabaGaemOBa4MaeyOeI0IaemOCaihaaaGcbaGaem4qam0aa0baaSqaaiabd6eaobqaaiabd6gaUbaaaaGccqGH9aqpdaWcaaqaaiabdoeadnaaDaaaleaacqWGUbGBaeaacqWGYbGCaaGccqGHflY1cqWGdbWqdaqhaaWcbaGaemOta4KaeyOeI0IaemOBa4gabaGaemOuaiLaeyOeI0IaemOCaihaaaGcbaGaem4qam0aa0baaSqaaiabd6eaobqaaiabdkfasbaaaaGccqGH9aqpdaWcaaqaaiabdkfasjabcgcaHiabcIcaOiabd6eaojabgkHiTiabdkfasjabcMcaPiabcgcaHaqaaiabd6eaojabcgcaHaaacqGHflY1daWcaaqaaiabd6gaUjabcgcaHiabcIcaOiabd6eaojabgkHiTiabd6gaUjabcMcaPiabcgcaHaqaaiabdkhaYjabcgcaHiabcIcaOiabdkfasjabgkHiTiabdkhaYjabcMcaPiabcgcaHaaacqGHflY1daWcaaqaaiabigdaXaqaaiabcIcaOiabd6gaUjabgkHiTiabdkhaYjabcMcaPiabcgcaHiabcIcaOiabd6eaojabgkHiTiabdkfasjabgkHiTiabd6gaUjabgUcaRiabdkhaYjabcMcaPiabcgcaHaaaaaa@8F41@

**N **represents all nodes in the MetaCore database of interactions. **R **is a subset a user's set of genes (**I**) that become "marked" because they correspond to the user's data. **n **is the number of nodes in a network/pathway module that is selected because of a common property, such as Gene Ontology category, set of nodes related to a certain disease, metabolic or signaling process, etc. **r **is the number of marked nodes among the **n **nodes in the module. The probability of a subset of size **n **to include **r **marked nodes, provided that **n **and **R **are unrelated (null-hypothesis), follows the hypergeometric distribution.

To assess the significance of the results other than microarray data, ANOVA and Student's t-test were employed. The error bars in the figures represent the standard error of the mean (SEM).

### Histology and immunohistochemistry

8 animals per time point with 5 sections per animal were analyzed. Sections were stained with hematoxylin and eosin, or immunohistochemically. Formalin-fixed sections were boiled in 10 mM Na-citrate pH 6.0 for 10 min to retrieve antigens. Monoclonal antibodies were directed against glutamine synthetase (GS/GLNS; Transduction Laboratories, Lexington, KY), proliferating-cell nuclear antigen (PCNA, Santa Cruz biotechnology, Santa Cruz, CA, USA) and smooth-muscle actin (α-SMA; Sigma, Zwijndrecht, The Netherlands), and polyclonal antibodies against carbamoylphosphate synthetase (CPS, [72]), active caspase 3 (CASP3; R&D Systems, Abingdon, United Kingdom) and phosphoenolpyruvate carboxykinase (PEPCK; kindly provided by Dr. Bruno Christ). Antibody binding was visualized with goat anti-mouse or goat anti-rabbit IgG, both coupled to alkaline phosphatase (Sigma). Goblet cells were visualized with Alcian blue. To quantify the tissue composition of the intestines, optical-density (OD) images of PCNA, CPS, GS, PEPCK and α-SMA stainings were analyzed by NIH Image software (ver.1.61, [73]). The PEPCK antibody did react with intestinal contents and stained the boundary between the epithelial cells and the intestinal lumen nonspecifically. This boundary was therefore not taken into account when quantifying the staining. The background was always subtracted in the measurements of light absorption in stained sections. The goblet and apoptotic cells were counted. The length of the villi was measured on well-oriented sections from the point of crypt-villus transition to the tip of the villus.

## Abbreviations

*Acadm *– α-keto acid dehydrogenase

*Acadyl *– acyl-coenzyme A dehydrogenase

*Adh1*/*5 *– alcohol dehydrogenases 1/5

*Akt1 *– thymoma viral proto-oncogene 1

*Aldh1a1 *– aldehyde dehydrogenase 1a1

ANOVA – analysis of variance

*Apaf1 *– apoptotic protease-activating factor 1

Asns – asparagine synthetase

*Atm1 *– ATP-binding cassette superfamily member of the mitochondrial inner membrane

*Bad *– Bcl-associated death promoter

BCAA – branched chain amino-acids

*Bcat1 *– branched-chain amino-acid transaminase

*Bcl2 *– B-cell leukaemia/lymphoma 2

*Bcl2l1*, *Bcl-xl *– Bcl2-like 1

*Casp1/6 *– caspase 1/6

*Ccng2 *– cyclin G2

*Cd36 *– fatty-acid transporter

*Cdk1/4 *– cyclin dependent kinase 1/4

*Cip1*, *p21 *– cyclin dependent kinase inhibitor 1

*Cps *– carbamoylphosphate synthetase

*Crot *– carnitine O-octanoyltransferase

*Cte1 *– cytosolic acyl-CoA thioesterase 1

CycA2 (B2, D, E1, H) – cyclin A2 (B2, D, E1, H)

*Dci *– dodecanoyl-Coenzyme A delta isomerase

dCTP – deoxy-cytosine triphosphate

*Fabp *– fatty acid biniding protein

FVB – mouse strain sensitive to Friend leukaemia virus B

*G6Pase *– glucose-6-phosphatase

*Gclc *– γ-glutamylcysteine synthetase

*Gln-ase, Gls *– glutaminase

*Glud *– glutamate dehydrogenase

*Got1 *– glutamate-oxaloacetate transaminase

*Gpd2 *– glycerolphosphate dehydrogenase 2

*Gs*, *Glns *– glutamine synthetase

*Gsk3b *– glycogen synthase kinase 3β

Gyk – glycerol kinase

*H2-Ab1 *– major histocompatibility group class II A-β1

HE – hematoxylin-eosin

*Hmgcs2 *– 3-hydroxy-3-methylglutaryl-Coenzyme A synthase 2

*Insig-1 *– insulin-induced protein 1

*Mod *– malic enzyme

NF – neurofilament

NFκBIA – nuclear factor of kappa light polypeptide gene enhancer in B-cells inhibitor, alpha

*Oat *– ornithine transaminase

OD – optical density

*Ogdh *– α-oxoglutarate dehydrogenase

*p53 *– transformation related protein 53

*Pcna *– proliferating cell nuclear antigen

*Pdk4 *– pyruvate dehydrogenase kinase 4

*Pepck, Pck1 *– phosphoenolpyruvate carboxykinase 1

*Pparα *– peroxisome proliferator-activated receptor, alpha isotype

*Prodh *– proline oxidase/dehydrogenase

qPCR – quantitative polymerase chain reaction

*Rb1 *– retinoblastoma

*Rbl1 *– retinoblastoma-like protein

RNA – ribonucleic acid

*Rxrα *– retinoid-X receptor α

*Scd1 *– stearoyl-Coenzyme A desaturase 1

*Sdha *– succinate dehydrogenase

SI – small intestine

*Sma *– α-smooth-muscle actin

TCA – tricarboxylic acid cycle

*Tpi *– triosephosphate isomerase

*UbC *– ubiquitin C

## Competing interests

The author(s) declares that there are no competing interests.

## Authors' contributions

MS carried out the biological part of the study and prepared the manuscript. AS and JV performed the morphological part of the research. DW and LGP designed and carried out the bioinformatics analysis of the data. AvK supervised this part of the study. RvH assisted with pathway analysis in GenMAPP environment, under supervision of CE. YN supported the data analysis in the MetaCore suite. TH and WL supervised the biological part of the study. All the authors read and approved the final manuscript.

## Supplementary Material

Additional file 1 Fold changes in response to fasting. The file provided contains lists of all the genes significantly regulated (≥ 1.4 fold) per time point of fasting. Click here for file

Additional file 2 Supplementary tables 1–3 Supplementary table 1 contains gene-specific primer sequences, product lengths, annealing temperatures, and MgCl_2 _concentrations. Supplementary table 2 contains a selection of genes with an expression pattern unique for a certain phase of fasting. Supplementary table 3 contains PPARα target genes differentially expressed in fasted intestine. Click here for file

Additional file 3 Pathway specific gene lists. The file contains lists of the genes significantly regulated in the pathways shown in the Figures 6–10. The data discussed in this publication have been deposited in NCBIs Gene Expression Omnibus (GEO, [74]) and are accessible through GEO Series accession number GSE8019. Click here for file
