# Supplementary material for: Fasting induces a biphasic adaptive metabolic response in murine small intestine
Source: BMC Genomics. 2007 Oct 9;8:361. doi: 10.1186/1471-2164-8-361 (PMC2148066; doi:10.1186/1471-2164-8-361)
Supplement: Additional file 2 — Supplementary tables 1–3 Supplementary table 1 contains gene-specific primer sequences, product lengths, annealing temperatures, and MgCl2 concentrations. Supplementary table 2 contains a selection of genes with an expression pattern unique for a certain phase of fasting. Supplementary table 3 contains PPARα target genes differentially expressed in fasted intestine. [file 1471-2164-8-361-S2.pdf]

Supplementary table 1: Gene-specific primer sequences, product lengths, annealing temperatures, and $MgCl_2$ concentrations.

**gene**	***primer sequence (5'→ 3')***	***product length (bp)***	***temp (°C)***	***$MgCl_2$ (mM)***
**18S**	F: TTCGGAACTGAGGCCATGAT<br>R: CGAACCTCCGACTTTCGTTCT	132	58	3
Casp6	F: GTGTTCGATCCAGCCGAG<br>R: GGCGTCGTATGCGTAAAC	309	55	3
Ccng 2	F: GCTAGCTTTGCATGCACCT<br>R: GCTCCATCACCACACAGAA	230	60	3
Gs	F: CCACCTCAGCAAGTTCCC<br>R: GGCTTCCGGTTATACTTG	318	55	3
Pck1	F: GCCAAGCTCACGCCCATC<br>R: CTCACGATTGTGCCGCTAT	329	58	3
Pdk4	F: GGGGGTGAACTGGTAGATTT<br>R: GCACCTTAGCTCTAGGTCA	191	58	3
H2-Ab1	F: GATCAAAGTGCGCTGGTT<br>R: GCCGCTCAACATCTTGCT	229	55	2
Rxrα	F: GCACGTACACCGGAACA<br>R: CGCTTCTAGTGACGCATA	217	53	3
UbC	F: CATCACCTTGGACGTCGA<br>R: AATGAAACTTGTTAACAGC	171	60	3

PCR primers were chosen at the 3' end of the molecule and spanned, when possible, exon-intron boundaries to avoid amplification of genomic DNA. 18S rRNA was used for normalization. Tested genes were: caspase 6 (*Casp6*), cyclin G2 (*Ccng2*), glutamine synthetase (*Gs*), phosphoenolpyruvate carboxykinase 1 (*Pck1*), pyruvate dehydrogenase kinase 4 (*Pdk4*), major histocompatibility group class II A-β1 (*H2-Ab1*), retinoid-X receptor α (*Rxrα*), ubiquitin C (*UbC*).

Supplementary table 2: Selection of genes with an expression pattern unique for a certain phase of fasting.

gene name	***description***	***fold change***		
short-term fasting		***12h***	***24h***	***72h***
Car1	carbonic anhydrase 1	4.2		
Acadvl	acyl-Coenzyme A dehydrogenase, very long chain	2.3		
Fabp4	fatty acid binding protein 4	2.2		
Slc34a2	solute carrier family 34 (sodium phosphate), member 2	5.4	1.9	
Des	desmin	3.0	3.9	
Gpx3	glutathione peroxidase 3	2.3	1.7	
Ogdh	oxoglutarate dehydrogenase (lipoamide)	2.1	1.8	
Ppara	peroxisome proliferator activated receptor alpha	1.4	2.2	
Cubn	cubilin (intrinsic factor-cobalamin receptor)	-3.0		
Gclm	glutamate-cysteine ligase, modifier subunit	-2.7		
prolonged fasting				
Ikbkg	inhibitor of kappaB kinase gamma			10.0
Creb3	cAMP responsive element binding protein 3			6.0
Timp2	tissue inhibitor of metalloproteinase 2			5.9
Rxra	retinoid X receptor alpha			5.5
Pcdh18	protocadherin 18			5.5
Jak3	Janus kinase 3			4.6
Lipc	lipase, hepatic			4.4
Cdkn1a	cyclin-dependent kinase inhibitor 1A (P21)			3.1
Dnclc1	dynein, cytoplasmic, light chain 1			-4.6
Tnxb	tenascin XB			-3.7

Shown are fold changes (increase represented by positive and decrease by negative numbers) of genes specifically up- or downregulated in short (12 and 24h) and prolonged fasting (72h), which could be used as biomarker collection.

Supplementary table 3: PPARα target genes are differentially expressed in fasted intestine.

gene symbol	***gene name***	***12h*** (*Ppara ↑*)	***24h*** (*Ppara ↑*)	***72h*** (*Ppara Ø*)
Crot	carnitine O-octanoyltransferase	-1.8	-1.7	-1.8
Cpt2	carnitine palmitoyltransferase 2	/	/	-3.3
Acadvl	acyl-Coenzyme A dehydrogenase, very long chain	2.3	/	/
Acadl	acyl-Coenzyme A dehydrogenase, long-chain	/	/	-1.8
Scd1	stearoyl-Coenzyme A desaturase 1	1.9	1.9	/
Dci	dodecanoyl-Coenzyme A delta isomerase (3,2 trans-enoyl-Coenzyme A isomerase)	1.4	/	-1.6
Mod1	malic enzyme, supernatant	-1.8	-1.5	-1.9
Hmgcs2	3-hydroxy-3-methylglutaryl-Coenzyme A synthase 2	3.8	2.2	2.6
Fabp4	fatty acid binding protein 4, adipocyte	2.2	/	/
Fabp5	fatty acid binding protein 5, epidermal	-1.6	/	-1.8
Gpd1	glycerol-3-phosphate dehydrogenase 1 (soluble)	/	/	-2.5
Gyk	glycerol kinase	/	-1.9	-1.7
Cte1	cytosolic acyl-CoA thioesterase 1	2.9	1.6	/
Cd36	CD36 antigen	/	/	2.1
Mgll	monoglyceride lipase	/	1.5	/
Pdk4	pyruvate dehydrogenase kinase, isoenzyme 4	2.5	2.4	7.3

The regulation of PPARα target genes (shown here as a fold change) coincides with a change in *Ppara* expression, depicted by symbols indicating its upregulation at 12 and 24h (1.4 and 2.2 fold respectively), and no change in expression at 72 hours of fasting.
